# [*N*′-(3-Meth­oxy-2-oxidobenzyl­idene)nicotinohydrazidato]diphenyl­tin(IV)

**DOI:** 10.1107/S1600536809033170

**Published:** 2009-08-26

**Authors:** Zhongjun Gao, Xiurong Zhai, Fangxia Zhou, Zhenjiao Cheng

**Affiliations:** a Department of Chemistry and Chemical Engineering, Jining University, Shandong 273155, People’s Republic of China

## Abstract

The asymmetric unit of the title compound, [Sn(C_6_H_5_)_2_(C_14_H_11_N_3_O_3_)], contains two crystallographically independent mol­ecules that differ predominantly in the torsion of the phenyl rings. In both mol­ecules, the Sn^IV^ ion is in a distored trigonal-bipyramidal geometry. The Sn—O distances are in the range 2.055 (2)–2.143 (2) Å.

## Related literature

For covalent radii see: Sanderson (1967 ▶). For a related structure see: Yearwood *et al.* (2002 ▶).
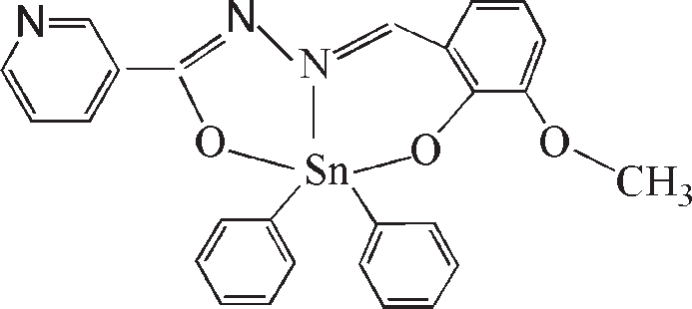


## Experimental

### 

#### Crystal data

[Sn(C_6_H_5_)_2_(C_14_H_11_N_3_O_3_)]
*M* *_r_* = 542.15
Monoclinic, 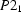

*a* = 8.9653 (14) Å
*b* = 20.771 (3) Å
*c* = 12.903 (2) Å
β = 106.015 (2)°
*V* = 2309.5 (6) Å^3^
*Z* = 4
Mo *K*α radiation
μ = 1.14 mm^−1^
*T* = 298 K
0.49 × 0.45 × 0.34 mm

#### Data collection

Bruker SMART CCD area-detector diffractometer
Absorption correction: multi-scan (*SADABS*; Sheldrick, 1996 ▶) *T*_min_ = 0.605, *T*_max_ = 0.698
9743 measured reflections
7970 independent reflections
7720 reflections with *I* > 2σ(*I*)
*R*_int_ = 0.019

#### Refinement

*R*[*F* ^2^ > 2σ(*F* ^2^)] = 0.024
*wR*(*F* ^2^) = 0.057
*S* = 1.02
7970 reflections
597 parameters
1 restraint
H-atom parameters constrained
Δρ_max_ = 0.26 e Å^−3^
Δρ_min_ = −0.49 e Å^−3^
Absolute structure: Flack (1983 ▶), 3753 Friedel pairs
Flack parameter: 0.0342

### 

Data collection: *SMART* (Siemens, 1996 ▶); cell refinement: *SAINT* (Siemens, 1996 ▶); data reduction: *SAINT*; program(s) used to solve structure: *SHELXS97* (Sheldrick, 2008 ▶); program(s) used to refine structure: *SHELXL97* (Sheldrick, 2008 ▶); molecular graphics: *SHELXTL* (Sheldrick, 2008 ▶); software used to prepare material for publication: *SHELXTL*.

## Supplementary Material

Crystal structure: contains datablocks I, global. DOI: 10.1107/S1600536809033170/nc2154sup1.cif

Structure factors: contains datablocks I. DOI: 10.1107/S1600536809033170/nc2154Isup2.hkl

Additional supplementary materials:  crystallographic information; 3D view; checkCIF report

**Fig. 1.**
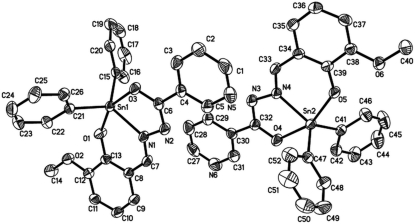
The molecular structure of the title complex with labeling and 30% displacement ellipsoids (H atoms omitted for clarity).

Enhanced figure: interactive version of Fig. 1

## Figures and Tables

**Table:** 

C15—Sn1	2.115 (3)
C21—Sn1	2.115 (3)
C41—Sn2	2.111 (3)
C47—Sn2	2.110 (3)
N1—Sn1	2.159 (3)
N4—Sn2	2.143 (3)
O1—Sn1	2.055 (2)
O3—Sn1	2.129 (2)
O4—Sn2	2.143 (2)
O5—Sn2	2.059 (2)

**Table:** 

O1—Sn1—C21	97.12 (12)
O1—Sn1—C15	94.22 (12)
C21—Sn1—C15	117.09 (12)
O1—Sn1—O3	156.71 (9)
C21—Sn1—O3	96.90 (11)
C15—Sn1—O3	95.83 (12)
O1—Sn1—N1	83.12 (11)
C21—Sn1—N1	121.10 (11)
C15—Sn1—N1	121.65 (12)
O3—Sn1—N1	73.72 (11)
O5—Sn2—C47	100.04 (12)
O5—Sn2—C41	95.16 (12)
C47—Sn2—C41	117.07 (13)
O5—Sn2—O4	155.61 (9)
C47—Sn2—O4	95.87 (11)
C41—Sn2—O4	93.89 (12)
O5—Sn2—N4	83.65 (11)
C47—Sn2—N4	112.89 (12)
C41—Sn2—N4	129.37 (12)
O4—Sn2—N4	73.13 (11)

## supplementary crystallographic information

### Comment

The structure determination was undertaken as a part of a project on the synthesis and structural characterization of Schiff base complexes. In the crystal structure of the title compound two crystallographically independent molecules are found. Differences are found predominantly in the torsion of the phenyl rings. The Sn1 atoms are five-coordinated by two O atoms, two C atoms and one N atom in a disorted trigonal-bipyramidal coodination (Table 1). The Schiff base is coordinated to the Sn1 atom as a tridentate ligand *via* the azomethine N atom, the hydroxyl O atom and the carbonyl O atom. The Sn1—N1 distances are close to the sum of the covalent radii of 2.15 Å (Sanderson, 1967), indicating a strong Sn—N interaction. Very similar structural parameters were observed in the compound studied by Yearwood *et al.* (2002).

### Experimental

A mixture of diphenyltin oxide (0.5778 g, 2.0 mmol) and 3-Methoxy-2-oxideobenzaldehyde(3-pyridyl)methyl-hydrazone (0.5420 g, 2.0 mmol) in methanol (60 ml) was heated under reflux for 6 h. The obtained clear solution was evaporated under vacuum. The product was crystallized from a mixture of dichloromethane/ethanol (1:1) to yield blocks of (I). Yield 0.8347 g, 77%, m.p. 476 K, analysis, calculated for C_26_H_21_N_3_O_3_Sn: C 57.60, H, 3.90; N 7.75%; found: C 57.66, H 3.93, N, 7.71%.

### Refinement

H atoms were positioned with idelaized geometry with C-H = 0.93 Å for aromatic H atoms and 0.96 Å for methyl H atoms and were constrained to ride on their parent atoms with *U*_iso_(H) = 1.2*U*_eq_(C) or 1.5*U*_eq_(for methyl groups). The absolute structure was determined on the basis of 3753 Friedel pairs.

### Crystal data

**Table:**

[Sn(C_6_H_5_)_2_(C_14_H_11_N_3_O_3_)]	*F*(000) = 1088
*M**_r_* = 542.15	*D*_x_ = 1.559 Mg m^−^^3^
Monoclinic, *P*2_1_	Mo *K*α radiation, λ = 0.71073 Å
Hall symbol: P 2yb	Cell parameters from 9021 reflections
*a* = 8.9653 (14) Å	θ = 2.4–28.1°
*b* = 20.771 (3) Å	µ = 1.14 mm^−^^1^
*c* = 12.903 (2) Å	*T* = 298 K
β = 106.015 (2)°	Block, yellow
*V* = 2309.5 (6) Å^3^	0.49 × 0.45 × 0.34 mm
*Z* = 4	

### Data collection

**Table:**

Bruker SMART CCD area-detector diffractometer	7970 independent reflections
Radiation source: fine-focus sealed tube	7720 reflections with *I* > 2σ(*I*)
graphite	*R*_int_ = 0.019
φ and ω scans	θ_max_ = 25.0°, θ_min_ = 1.9°
Absorption correction: multi-scan (*SADABS*; Sheldrick, 1996)	*h* = −10→10
*T*_min_ = 0.605, *T*_max_ = 0.698	*k* = −24→23
9743 measured reflections	*l* = −15→9

### Refinement

**Table:**

Refinement on *F*^2^	Secondary atom site location: difference Fourier map
Least-squares matrix: full	Hydrogen site location: inferred from neighbouring sites
*R*[*F*^2^ > 2σ(*F*^2^)] = 0.024	H-atom parameters constrained
*wR*(*F*^2^) = 0.057	*w* = 1/[σ^2^(*F*_o_^2^) + (0.0304*P*)^2^] where *P* = (*F*_o_^2^ + 2*F*_c_^2^)/3
*S* = 1.02	(Δ/σ)_max_ = 0.001
7970 reflections	Δρ_max_ = 0.26 e Å^−^^3^
597 parameters	Δρ_min_ = −0.49 e Å^−^^3^
1 restraint	Absolute structure: Flack (1983), 3753 Friedel pairs
Primary atom site location: structure-invariant direct methods	Flack parameter: 0.0342

### Special details

**Table:**

Geometry. All e.s.d.'s (except the e.s.d. in the dihedral angle between two l.s. planes) are estimated using the full covariance matrix. The cell e.s.d.'s are taken into account individually in the estimation of e.s.d.'s in distances, angles and torsion angles; correlations between e.s.d.'s in cell parameters are only used when they are defined by crystal symmetry. An approximate (isotropic) treatment of cell e.s.d.'s is used for estimating e.s.d.'s involving l.s. planes.
Refinement. Refinement of *F*^2^ against ALL reflections. The weighted *R*-factor *wR* and goodness of fit *S* are based on *F*^2^, conventional *R*-factors *R* are based on *F*, with *F* set to zero for negative *F*^2^. The threshold expression of *F*^2^ > σ(*F*^2^) is used only for calculating *R*-factors(gt) *etc*. and is not relevant to the choice of reflections for refinement. *R*-factors based on *F*^2^ are statistically about twice as large as those based on *F*, and *R*- factors based on ALL data will be even larger.

### Fractional atomic coordinates and isotropic or equivalent isotropic displacement parameters (Å^2^)

**Table:**

	*x*	*y*	*z*	*U*_iso_*/*U*_eq_	
C1	0.8744 (5)	0.6897 (3)	−0.3633 (3)	0.0776 (13)	
H1	0.9124	0.6724	−0.4174	0.093*	
C2	0.8341 (5)	0.6481 (2)	−0.2940 (3)	0.0701 (11)	
H2	0.8451	0.6038	−0.3000	0.084*	
C3	0.7773 (5)	0.67369 (19)	−0.2162 (3)	0.0616 (10)	
H3	0.7483	0.6464	−0.1680	0.074*	
C4	0.7615 (4)	0.73871 (18)	−0.2066 (3)	0.0464 (8)	
C5	0.8047 (5)	0.7776 (2)	−0.2807 (3)	0.0636 (9)	
H5	0.7928	0.8219	−0.2765	0.076*	
C6	0.7003 (3)	0.76720 (19)	−0.1211 (2)	0.0439 (7)	
C7	0.6190 (4)	0.9109 (2)	−0.0230 (3)	0.0493 (8)	
H7	0.6455	0.9369	−0.0739	0.059*	
C8	0.5591 (4)	0.94252 (17)	0.0557 (3)	0.0450 (7)	
C9	0.5271 (5)	1.00873 (19)	0.0427 (3)	0.0617 (10)	
H9	0.5505	1.0306	−0.0137	0.074*	
C10	0.4628 (5)	1.04139 (19)	0.1105 (3)	0.0663 (11)	
H10	0.4433	1.0853	0.1008	0.080*	
C11	0.4258 (4)	1.00908 (19)	0.1950 (3)	0.0562 (9)	
H11	0.3798	1.0315	0.2405	0.067*	
C12	0.4566 (4)	0.94435 (17)	0.2120 (3)	0.0451 (8)	
C13	0.5281 (3)	0.9105 (2)	0.1433 (2)	0.0423 (7)	
C14	0.3611 (6)	0.9384 (2)	0.3658 (3)	0.0778 (14)	
H14A	0.4295	0.9713	0.4040	0.117*	
H14B	0.3445	0.9070	0.4161	0.117*	
H14C	0.2636	0.9572	0.3278	0.117*	
C15	0.7839 (4)	0.73385 (18)	0.1970 (3)	0.0467 (8)	
C16	0.8784 (4)	0.7704 (3)	0.2785 (3)	0.0642 (10)	
H16	0.8672	0.8150	0.2783	0.077*	
C17	0.9909 (5)	0.7405 (3)	0.3613 (3)	0.0903 (17)	
H17	1.0556	0.7650	0.4158	0.108*	
C18	1.0053 (6)	0.6746 (4)	0.3617 (4)	0.0964 (19)	
H18	1.0799	0.6547	0.4170	0.116*	
C19	0.9121 (5)	0.6382 (3)	0.2823 (4)	0.0799 (13)	
H19	0.9218	0.5936	0.2838	0.096*	
C20	0.8033 (4)	0.6679 (2)	0.1997 (3)	0.0619 (10)	
H20	0.7415	0.6429	0.1446	0.074*	
C21	0.3786 (4)	0.73152 (16)	0.0491 (2)	0.0431 (7)	
C22	0.2814 (4)	0.75321 (18)	0.1083 (3)	0.0557 (9)	
H22	0.3108	0.7879	0.1550	0.067*	
C23	0.1402 (5)	0.7230 (2)	0.0975 (4)	0.0725 (12)	
H23	0.0753	0.7372	0.1380	0.087*	
C24	0.0961 (5)	0.6727 (2)	0.0281 (4)	0.0723 (12)	
H24	0.0005	0.6530	0.0205	0.087*	
C25	0.1911 (5)	0.6512 (2)	−0.0301 (3)	0.0703 (11)	
H25	0.1605	0.6169	−0.0775	0.084*	
C26	0.3332 (4)	0.68023 (18)	−0.0189 (3)	0.0525 (8)	
H26	0.3988	0.6648	−0.0579	0.063*	
C27	0.0013 (5)	0.9828 (3)	1.1151 (4)	0.0785 (14)	
H27	−0.0341	0.9980	1.1719	0.094*	
C28	0.0037 (5)	0.9185 (3)	1.1010 (3)	0.0764 (11)	
H28	−0.0280	0.8907	1.1474	0.092*	
C29	0.0535 (5)	0.8947 (2)	1.0178 (3)	0.0643 (11)	
H29	0.0553	0.8506	1.0061	0.077*	
C30	0.1012 (4)	0.93724 (18)	0.9516 (3)	0.0499 (8)	
C31	0.0949 (5)	1.0015 (2)	0.9724 (3)	0.0693 (11)	
H31	0.1268	1.0302	0.9274	0.083*	
C32	0.1608 (4)	0.9145 (2)	0.8613 (2)	0.0474 (7)	
C33	0.2466 (4)	0.7762 (2)	0.7514 (2)	0.0496 (8)	
H33	0.2118	0.7488	0.7968	0.060*	
C34	0.3089 (4)	0.74560 (17)	0.6728 (3)	0.0457 (8)	
C35	0.3156 (5)	0.67813 (19)	0.6717 (3)	0.0589 (9)	
H35	0.2772	0.6547	0.7202	0.071*	
C36	0.3774 (5)	0.64637 (19)	0.6007 (3)	0.0630 (10)	
H36	0.3809	0.6016	0.6008	0.076*	
C37	0.4350 (5)	0.68106 (19)	0.5283 (3)	0.0565 (9)	
H37	0.4777	0.6594	0.4802	0.068*	
C38	0.4295 (4)	0.74709 (17)	0.5272 (3)	0.0478 (8)	
C39	0.3666 (4)	0.78110 (17)	0.6002 (2)	0.0443 (7)	
C40	0.5481 (6)	0.7553 (2)	0.3835 (3)	0.0762 (13)	
H40A	0.6348	0.7293	0.4207	0.114*	
H40B	0.5823	0.7875	0.3418	0.114*	
H40C	0.4711	0.7286	0.3364	0.114*	
C41	0.4735 (4)	0.97590 (17)	0.6996 (3)	0.0476 (8)	
C42	0.4710 (5)	1.03547 (19)	0.7470 (3)	0.0603 (10)	
H42	0.3837	1.0477	0.7678	0.072*	
C43	0.5954 (6)	1.0772 (2)	0.7640 (4)	0.0797 (13)	
H43	0.5916	1.1172	0.7955	0.096*	
C44	0.7229 (6)	1.0592 (3)	0.7345 (5)	0.0973 (17)	
H44	0.8071	1.0870	0.7462	0.117*	
C45	0.7294 (6)	1.0005 (3)	0.6876 (5)	0.0980 (17)	
H45	0.8182	0.9886	0.6684	0.118*	
C46	0.6038 (5)	0.9588 (2)	0.6685 (3)	0.0699 (11)	
H46	0.6073	0.9194	0.6350	0.084*	
C47	0.0803 (4)	0.94225 (17)	0.5471 (3)	0.0460 (7)	
C48	0.0773 (5)	1.0006 (2)	0.4981 (3)	0.0672 (11)	
H48	0.1631	1.0276	0.5197	0.081*	
C49	−0.0503 (6)	1.0207 (3)	0.4169 (4)	0.0865 (15)	
H49	−0.0515	1.0612	0.3859	0.104*	
C50	−0.1721 (5)	0.9809 (3)	0.3834 (4)	0.0833 (14)	
H50	−0.2568	0.9937	0.3275	0.100*	
C51	−0.1740 (5)	0.9226 (3)	0.4294 (4)	0.0919 (15)	
H51	−0.2597	0.8958	0.4058	0.110*	
C52	−0.0482 (5)	0.9032 (2)	0.5116 (4)	0.0753 (13)	
H52	−0.0499	0.8632	0.5436	0.090*	
N1	0.6398 (3)	0.84948 (14)	−0.0300 (2)	0.0438 (6)	
N2	0.6926 (4)	0.82985 (15)	−0.1167 (2)	0.0502 (7)	
N3	0.1728 (4)	0.85239 (16)	0.8523 (2)	0.0571 (8)	
N4	0.2328 (3)	0.83689 (15)	0.7669 (2)	0.0473 (7)	
N5	0.8630 (5)	0.7541 (2)	−0.3585 (3)	0.0853 (13)	
N6	0.0461 (5)	1.0262 (2)	1.0533 (3)	0.0824 (11)	
O1	0.5663 (3)	0.84980 (12)	0.16702 (17)	0.0551 (6)	
O2	0.4289 (3)	0.90798 (15)	0.29086 (18)	0.0591 (7)	
O3	0.6595 (3)	0.72733 (11)	−0.05616 (18)	0.0519 (6)	
O4	0.1992 (3)	0.95651 (12)	0.8002 (2)	0.0596 (7)	
O5	0.3664 (3)	0.84455 (12)	0.5949 (2)	0.0608 (7)	
O6	0.4834 (3)	0.78554 (13)	0.4596 (2)	0.0646 (7)	
Sn1	0.60021 (2)	0.773826 (10)	0.074059 (15)	0.03996 (6)	
Sn2	0.27721 (3)	0.915473 (10)	0.672005 (17)	0.04469 (7)	

### Atomic displacement parameters (Å^2^)

**Table:**

	*U*^11^	*U*^22^	*U*^33^	*U*^12^	*U*^13^	*U*^23^
C1	0.085 (3)	0.100 (4)	0.061 (2)	0.023 (3)	0.042 (2)	−0.005 (2)
C2	0.094 (3)	0.064 (3)	0.064 (2)	0.010 (2)	0.043 (2)	−0.008 (2)
C3	0.082 (3)	0.056 (2)	0.057 (2)	−0.008 (2)	0.039 (2)	−0.0040 (17)
C4	0.0432 (17)	0.060 (2)	0.0395 (17)	−0.0020 (16)	0.0167 (14)	−0.0014 (15)
C5	0.082 (2)	0.061 (2)	0.059 (2)	0.009 (2)	0.0384 (18)	0.009 (2)
C6	0.0449 (16)	0.055 (2)	0.0354 (15)	−0.0008 (17)	0.0168 (12)	−0.0034 (16)
C7	0.061 (2)	0.047 (2)	0.0442 (17)	−0.0035 (19)	0.0220 (14)	0.0087 (17)
C8	0.0499 (18)	0.0440 (19)	0.0437 (17)	0.0006 (15)	0.0172 (14)	0.0031 (14)
C9	0.079 (3)	0.051 (2)	0.066 (2)	0.008 (2)	0.038 (2)	0.0156 (17)
C10	0.088 (3)	0.042 (2)	0.080 (3)	0.010 (2)	0.041 (2)	0.0101 (18)
C11	0.062 (2)	0.053 (2)	0.059 (2)	0.0109 (18)	0.0256 (18)	−0.0009 (17)
C12	0.0495 (19)	0.0454 (19)	0.0402 (17)	−0.0005 (15)	0.0120 (14)	−0.0025 (14)
C13	0.0419 (16)	0.0421 (19)	0.0403 (16)	0.0008 (16)	0.0071 (12)	0.0020 (17)
C14	0.115 (4)	0.075 (3)	0.059 (2)	0.025 (3)	0.050 (2)	0.006 (2)
C15	0.0395 (17)	0.062 (2)	0.0421 (17)	0.0066 (15)	0.0179 (13)	0.0091 (16)
C16	0.056 (2)	0.084 (3)	0.053 (2)	0.003 (2)	0.0153 (16)	−0.009 (2)
C17	0.058 (3)	0.159 (6)	0.049 (2)	0.005 (3)	0.007 (2)	−0.018 (3)
C18	0.068 (3)	0.152 (6)	0.072 (3)	0.044 (4)	0.022 (3)	0.035 (4)
C19	0.067 (3)	0.091 (3)	0.086 (3)	0.027 (3)	0.027 (2)	0.032 (3)
C20	0.050 (2)	0.069 (3)	0.072 (2)	0.0092 (18)	0.0244 (18)	0.019 (2)
C21	0.0407 (16)	0.0498 (19)	0.0387 (16)	0.0030 (14)	0.0107 (13)	0.0099 (14)
C22	0.0511 (19)	0.060 (2)	0.062 (2)	0.0048 (16)	0.0253 (17)	−0.0002 (16)
C23	0.056 (2)	0.084 (3)	0.087 (3)	0.008 (2)	0.036 (2)	0.008 (3)
C24	0.050 (2)	0.087 (3)	0.082 (3)	−0.013 (2)	0.022 (2)	0.012 (3)
C25	0.073 (3)	0.074 (3)	0.062 (2)	−0.025 (2)	0.015 (2)	−0.004 (2)
C26	0.060 (2)	0.057 (2)	0.0459 (18)	−0.0020 (17)	0.0219 (16)	−0.0008 (16)
C27	0.070 (3)	0.110 (4)	0.062 (3)	0.025 (3)	0.030 (2)	−0.009 (3)
C28	0.079 (3)	0.099 (4)	0.062 (2)	0.003 (3)	0.037 (2)	0.003 (3)
C29	0.073 (3)	0.067 (3)	0.062 (2)	0.004 (2)	0.034 (2)	0.0059 (19)
C30	0.0490 (19)	0.060 (2)	0.0419 (18)	0.0080 (16)	0.0142 (15)	0.0009 (15)
C31	0.085 (3)	0.065 (3)	0.067 (3)	0.012 (2)	0.038 (2)	0.005 (2)
C32	0.0539 (18)	0.0483 (19)	0.0430 (16)	0.0005 (19)	0.0186 (14)	0.0032 (18)
C33	0.064 (2)	0.0441 (19)	0.0452 (17)	−0.005 (2)	0.0219 (14)	0.0073 (18)
C34	0.0499 (18)	0.0388 (18)	0.0484 (19)	0.0013 (15)	0.0135 (15)	0.0012 (13)
C35	0.076 (3)	0.047 (2)	0.060 (2)	−0.0009 (18)	0.0286 (19)	0.0061 (17)
C36	0.080 (3)	0.039 (2)	0.075 (3)	0.0052 (19)	0.029 (2)	0.0006 (18)
C37	0.067 (2)	0.050 (2)	0.054 (2)	0.0110 (18)	0.0191 (18)	−0.0052 (17)
C38	0.051 (2)	0.0458 (19)	0.0454 (19)	0.0032 (15)	0.0121 (15)	0.0017 (14)
C39	0.0524 (18)	0.0377 (18)	0.0407 (16)	0.0022 (16)	0.0093 (13)	0.0019 (15)
C40	0.108 (4)	0.076 (3)	0.057 (2)	0.017 (3)	0.043 (2)	0.001 (2)
C41	0.055 (2)	0.0483 (19)	0.0407 (17)	−0.0021 (15)	0.0143 (14)	0.0044 (14)
C42	0.067 (2)	0.053 (2)	0.068 (2)	−0.0056 (19)	0.030 (2)	−0.0070 (18)
C43	0.097 (3)	0.071 (3)	0.075 (3)	−0.028 (3)	0.031 (3)	−0.013 (2)
C44	0.088 (4)	0.104 (4)	0.110 (4)	−0.045 (3)	0.045 (3)	−0.031 (3)
C45	0.068 (3)	0.121 (5)	0.119 (4)	−0.021 (3)	0.049 (3)	−0.032 (4)
C46	0.067 (3)	0.072 (3)	0.076 (3)	−0.001 (2)	0.029 (2)	−0.012 (2)
C47	0.0522 (19)	0.0438 (18)	0.0445 (17)	0.0013 (15)	0.0177 (14)	−0.0047 (14)
C48	0.062 (2)	0.061 (2)	0.070 (3)	−0.007 (2)	0.0053 (19)	0.017 (2)
C49	0.079 (3)	0.078 (3)	0.088 (3)	0.004 (3)	−0.002 (3)	0.027 (3)
C50	0.067 (3)	0.083 (4)	0.085 (3)	0.012 (3)	−0.005 (2)	−0.018 (3)
C51	0.068 (3)	0.077 (3)	0.115 (4)	−0.017 (3)	−0.001 (3)	−0.039 (3)
C52	0.066 (3)	0.055 (3)	0.100 (3)	−0.014 (2)	0.016 (2)	−0.014 (2)
N1	0.0552 (17)	0.0433 (17)	0.0377 (14)	−0.0021 (13)	0.0209 (12)	0.0029 (12)
N2	0.0674 (19)	0.0473 (19)	0.0445 (16)	0.0007 (15)	0.0301 (14)	0.0017 (13)
N3	0.074 (2)	0.054 (2)	0.0528 (18)	0.0053 (16)	0.0345 (16)	0.0043 (15)
N4	0.0607 (18)	0.0429 (18)	0.0456 (17)	0.0028 (14)	0.0268 (13)	0.0037 (13)
N5	0.104 (3)	0.100 (3)	0.075 (2)	0.021 (2)	0.063 (2)	0.013 (2)
N6	0.104 (3)	0.077 (3)	0.078 (2)	0.022 (2)	0.045 (2)	−0.008 (2)
O1	0.0940 (19)	0.0379 (13)	0.0410 (12)	0.0060 (13)	0.0315 (12)	−0.0007 (11)
O2	0.0863 (17)	0.0531 (16)	0.0475 (12)	0.0106 (15)	0.0347 (12)	0.0042 (12)
O3	0.0746 (16)	0.0436 (13)	0.0481 (14)	−0.0028 (12)	0.0350 (12)	−0.0007 (11)
O4	0.0888 (19)	0.0440 (14)	0.0577 (15)	0.0000 (13)	0.0397 (14)	0.0001 (12)
O5	0.099 (2)	0.0379 (14)	0.0622 (16)	0.0050 (13)	0.0495 (14)	0.0031 (12)
O6	0.0962 (19)	0.0523 (17)	0.0597 (15)	0.0075 (14)	0.0455 (14)	0.0014 (12)
Sn1	0.04690 (12)	0.04038 (12)	0.03702 (11)	0.00066 (11)	0.01897 (8)	0.00278 (10)
Sn2	0.05794 (14)	0.03805 (12)	0.04156 (12)	0.00031 (11)	0.01956 (10)	0.00249 (10)

### Geometric parameters (Å, °)

**Table:**

C1—N5	1.345 (6)	C28—H28	0.9300
C1—C2	1.361 (6)	C29—C30	1.377 (5)
C1—H1	0.9300	C29—H29	0.9300
C2—C3	1.354 (5)	C30—C31	1.367 (5)
C2—H2	0.9300	C30—C32	1.487 (5)
C3—C4	1.367 (5)	C31—N6	1.340 (5)
C3—H3	0.9300	C31—H31	0.9300
C4—C5	1.386 (5)	C32—O4	1.285 (4)
C4—C6	1.485 (4)	C32—N3	1.302 (5)
C5—N5	1.345 (5)	C33—N4	1.288 (6)
C5—H5	0.9300	C33—C34	1.434 (5)
C6—O3	1.300 (4)	C33—H33	0.9300
C6—N2	1.305 (5)	C34—C39	1.399 (5)
C7—N1	1.296 (5)	C34—C35	1.403 (5)
C7—C8	1.432 (5)	C35—C36	1.365 (5)
C7—H7	0.9300	C35—H35	0.9300
C8—C13	1.404 (5)	C36—C37	1.387 (5)
C8—C9	1.405 (5)	C36—H36	0.9300
C9—C10	1.355 (5)	C37—C38	1.372 (5)
C9—H9	0.9300	C37—H37	0.9300
C10—C11	1.396 (5)	C38—O6	1.366 (4)
C10—H10	0.9300	C38—C39	1.413 (5)
C11—C12	1.378 (5)	C39—O5	1.320 (4)
C11—H11	0.9300	C40—O6	1.417 (4)
C12—O2	1.344 (4)	C40—H40A	0.9600
C12—C13	1.416 (5)	C40—H40B	0.9600
C13—O1	1.321 (5)	C40—H40C	0.9600
C14—O2	1.424 (4)	C41—C46	1.382 (5)
C14—H14A	0.9600	C41—C42	1.383 (5)
C14—H14B	0.9600	C41—Sn2	2.111 (3)
C14—H14C	0.9600	C42—C43	1.381 (6)
C15—C20	1.381 (6)	C42—H42	0.9300
C15—C16	1.383 (5)	C43—C44	1.354 (7)
C15—Sn1	2.115 (3)	C43—H43	0.9300
C16—C17	1.396 (7)	C44—C45	1.370 (8)
C16—H16	0.9300	C44—H44	0.9300
C17—C18	1.374 (8)	C45—C46	1.388 (7)
C17—H17	0.9300	C45—H45	0.9300
C18—C19	1.359 (8)	C46—H46	0.9300
C18—H18	0.9300	C47—C48	1.364 (5)
C19—C20	1.375 (6)	C47—C52	1.380 (5)
C19—H19	0.9300	C47—Sn2	2.110 (3)
C20—H20	0.9300	C48—C49	1.386 (6)
C21—C26	1.369 (5)	C48—H48	0.9300
C21—C22	1.382 (5)	C49—C50	1.342 (7)
C21—Sn1	2.115 (3)	C49—H49	0.9300
C22—C23	1.385 (6)	C50—C51	1.351 (8)
C22—H22	0.9300	C50—H50	0.9300
C23—C24	1.363 (7)	C51—C52	1.379 (7)
C23—H23	0.9300	C51—H51	0.9300
C24—C25	1.357 (6)	C52—H52	0.9300
C24—H24	0.9300	N1—N2	1.390 (4)
C25—C26	1.381 (5)	N1—Sn1	2.159 (3)
C25—H25	0.9300	N3—N4	1.390 (4)
C26—H26	0.9300	N4—Sn2	2.143 (3)
C27—N6	1.337 (6)	O1—Sn1	2.055 (2)
C27—C28	1.350 (8)	O3—Sn1	2.129 (2)
C27—H27	0.9300	O4—Sn2	2.143 (2)
C28—C29	1.364 (6)	O5—Sn2	2.059 (2)
			
N5—C1—C2	124.3 (4)	N4—C33—C34	128.1 (3)
N5—C1—H1	117.8	N4—C33—H33	116.0
C2—C1—H1	117.8	C34—C33—H33	116.0
C3—C2—C1	117.4 (4)	C39—C34—C35	119.7 (3)
C3—C2—H2	121.3	C39—C34—C33	121.9 (3)
C1—C2—H2	121.3	C35—C34—C33	118.4 (3)
C2—C3—C4	121.6 (4)	C36—C35—C34	121.0 (4)
C2—C3—H3	119.2	C36—C35—H35	119.5
C4—C3—H3	119.2	C34—C35—H35	119.5
C3—C4—C5	117.3 (3)	C35—C36—C37	119.8 (4)
C3—C4—C6	122.0 (3)	C35—C36—H36	120.1
C5—C4—C6	120.7 (4)	C37—C36—H36	120.1
N5—C5—C4	122.9 (5)	C38—C37—C36	120.5 (4)
N5—C5—H5	118.6	C38—C37—H37	119.8
C4—C5—H5	118.6	C36—C37—H37	119.8
O3—C6—N2	125.4 (3)	O6—C38—C37	125.0 (3)
O3—C6—C4	116.9 (3)	O6—C38—C39	114.2 (3)
N2—C6—C4	117.7 (3)	C37—C38—C39	120.8 (3)
N1—C7—C8	126.1 (3)	O5—C39—C34	124.4 (3)
N1—C7—H7	116.9	O5—C39—C38	117.4 (3)
C8—C7—H7	116.9	C34—C39—C38	118.2 (3)
C13—C8—C9	118.7 (3)	O6—C40—H40A	109.5
C13—C8—C7	123.4 (4)	O6—C40—H40B	109.5
C9—C8—C7	117.9 (3)	H40A—C40—H40B	109.5
C10—C9—C8	121.5 (3)	O6—C40—H40C	109.5
C10—C9—H9	119.3	H40A—C40—H40C	109.5
C8—C9—H9	119.3	H40B—C40—H40C	109.5
C9—C10—C11	119.9 (4)	C46—C41—C42	118.5 (4)
C9—C10—H10	120.0	C46—C41—Sn2	122.0 (3)
C11—C10—H10	120.0	C42—C41—Sn2	119.5 (3)
C12—C11—C10	120.9 (3)	C43—C42—C41	121.4 (4)
C12—C11—H11	119.6	C43—C42—H42	119.3
C10—C11—H11	119.6	C41—C42—H42	119.3
O2—C12—C11	126.7 (3)	C44—C43—C42	119.3 (5)
O2—C12—C13	113.9 (3)	C44—C43—H43	120.3
C11—C12—C13	119.3 (3)	C42—C43—H43	120.3
O1—C13—C8	122.8 (3)	C43—C44—C45	120.8 (5)
O1—C13—C12	117.5 (3)	C43—C44—H44	119.6
C8—C13—C12	119.6 (4)	C45—C44—H44	119.6
O2—C14—H14A	109.5	C44—C45—C46	120.2 (5)
O2—C14—H14B	109.5	C44—C45—H45	119.9
H14A—C14—H14B	109.5	C46—C45—H45	119.9
O2—C14—H14C	109.5	C41—C46—C45	119.8 (4)
H14A—C14—H14C	109.5	C41—C46—H46	120.1
H14B—C14—H14C	109.5	C45—C46—H46	120.1
C20—C15—C16	118.7 (4)	C48—C47—C52	117.5 (4)
C20—C15—Sn1	118.4 (3)	C48—C47—Sn2	119.6 (3)
C16—C15—Sn1	122.8 (3)	C52—C47—Sn2	123.0 (3)
C15—C16—C17	119.9 (5)	C47—C48—C49	121.7 (4)
C15—C16—H16	120.0	C47—C48—H48	119.2
C17—C16—H16	120.0	C49—C48—H48	119.2
C18—C17—C16	119.6 (5)	C50—C49—C48	119.1 (5)
C18—C17—H17	120.2	C50—C49—H49	120.4
C16—C17—H17	120.2	C48—C49—H49	120.4
C19—C18—C17	120.9 (5)	C49—C50—C51	121.2 (4)
C19—C18—H18	119.6	C49—C50—H50	119.4
C17—C18—H18	119.6	C51—C50—H50	119.4
C18—C19—C20	119.5 (5)	C50—C51—C52	119.6 (4)
C18—C19—H19	120.3	C50—C51—H51	120.2
C20—C19—H19	120.3	C52—C51—H51	120.2
C19—C20—C15	121.5 (5)	C51—C52—C47	120.9 (5)
C19—C20—H20	119.3	C51—C52—H52	119.5
C15—C20—H20	119.3	C47—C52—H52	119.5
C26—C21—C22	119.1 (3)	C7—N1—N2	115.6 (3)
C26—C21—Sn1	121.4 (2)	C7—N1—Sn1	128.4 (2)
C22—C21—Sn1	119.3 (3)	N2—N1—Sn1	115.9 (2)
C21—C22—C23	119.7 (4)	C6—N2—N1	111.2 (3)
C21—C22—H22	120.2	C32—N3—N4	111.1 (3)
C23—C22—H22	120.2	C33—N4—N3	115.1 (3)
C24—C23—C22	120.3 (4)	C33—N4—Sn2	128.0 (2)
C24—C23—H23	119.8	N3—N4—Sn2	116.7 (2)
C22—C23—H23	119.8	C1—N5—C5	116.5 (4)
C25—C24—C23	120.2 (4)	C27—N6—C31	115.0 (4)
C25—C24—H24	119.9	C13—O1—Sn1	131.97 (19)
C23—C24—H24	119.9	C12—O2—C14	117.9 (3)
C24—C25—C26	120.0 (4)	C6—O3—Sn1	113.3 (2)
C24—C25—H25	120.0	C32—O4—Sn2	113.7 (2)
C26—C25—H25	120.0	C39—O5—Sn2	133.2 (2)
C21—C26—C25	120.6 (4)	C38—O6—C40	117.9 (3)
C21—C26—H26	119.7	O1—Sn1—C21	97.12 (12)
C25—C26—H26	119.7	O1—Sn1—C15	94.22 (12)
N6—C27—C28	124.7 (4)	C21—Sn1—C15	117.09 (12)
N6—C27—H27	117.7	O1—Sn1—O3	156.71 (9)
C28—C27—H27	117.7	C21—Sn1—O3	96.90 (11)
C27—C28—C29	119.0 (5)	C15—Sn1—O3	95.83 (12)
C27—C28—H28	120.5	O1—Sn1—N1	83.12 (11)
C29—C28—H28	120.5	C21—Sn1—N1	121.10 (11)
C28—C29—C30	118.8 (4)	C15—Sn1—N1	121.65 (12)
C28—C29—H29	120.6	O3—Sn1—N1	73.72 (11)
C30—C29—H29	120.6	O5—Sn2—C47	100.04 (12)
C31—C30—C29	117.9 (4)	O5—Sn2—C41	95.16 (12)
C31—C30—C32	120.6 (4)	C47—Sn2—C41	117.07 (13)
C29—C30—C32	121.5 (4)	O5—Sn2—O4	155.61 (9)
N6—C31—C30	124.5 (4)	C47—Sn2—O4	95.87 (11)
N6—C31—H31	117.7	C41—Sn2—O4	93.89 (12)
C30—C31—H31	117.7	O5—Sn2—N4	83.65 (11)
O4—C32—N3	125.2 (3)	C47—Sn2—N4	112.89 (12)
O4—C32—C30	118.6 (4)	C41—Sn2—N4	129.37 (12)
N3—C32—C30	116.2 (3)	O4—Sn2—N4	73.13 (11)
			
N5—C1—C2—C3	0.7 (8)	C7—N1—N2—C6	−175.9 (3)
C1—C2—C3—C4	−0.3 (7)	Sn1—N1—N2—C6	3.4 (4)
C2—C3—C4—C5	0.6 (6)	O4—C32—N3—N4	−0.2 (5)
C2—C3—C4—C6	−179.6 (4)	C30—C32—N3—N4	−178.6 (3)
C3—C4—C5—N5	−1.3 (6)	C34—C33—N4—N3	−177.8 (3)
C6—C4—C5—N5	178.9 (4)	C34—C33—N4—Sn2	6.4 (5)
C3—C4—C6—O3	−1.1 (5)	C32—N3—N4—C33	−179.1 (3)
C5—C4—C6—O3	178.6 (3)	C32—N3—N4—Sn2	−2.8 (4)
C3—C4—C6—N2	178.5 (4)	C2—C1—N5—C5	−1.4 (8)
C5—C4—C6—N2	−1.8 (5)	C4—C5—N5—C1	1.7 (7)
N1—C7—C8—C13	6.3 (6)	C28—C27—N6—C31	0.4 (7)
N1—C7—C8—C9	−172.4 (4)	C30—C31—N6—C27	−0.2 (7)
C13—C8—C9—C10	−2.1 (6)	C8—C13—O1—Sn1	−25.6 (5)
C7—C8—C9—C10	176.6 (4)	C12—C13—O1—Sn1	156.2 (2)
C8—C9—C10—C11	−0.5 (7)	C11—C12—O2—C14	−0.1 (6)
C9—C10—C11—C12	1.3 (6)	C13—C12—O2—C14	178.3 (3)
C10—C11—C12—O2	179.0 (4)	N2—C6—O3—Sn1	−6.7 (4)
C10—C11—C12—C13	0.6 (6)	C4—C6—O3—Sn1	172.9 (2)
C9—C8—C13—O1	−174.2 (3)	N3—C32—O4—Sn2	3.0 (4)
C7—C8—C13—O1	7.2 (5)	C30—C32—O4—Sn2	−178.6 (2)
C9—C8—C13—C12	4.0 (5)	C34—C39—O5—Sn2	−5.2 (5)
C7—C8—C13—C12	−174.6 (3)	C38—C39—O5—Sn2	174.8 (2)
O2—C12—C13—O1	−3.6 (4)	C37—C38—O6—C40	1.0 (6)
C11—C12—C13—O1	175.0 (3)	C39—C38—O6—C40	−179.9 (3)
O2—C12—C13—C8	178.1 (3)	C13—O1—Sn1—C21	−98.2 (3)
C11—C12—C13—C8	−3.3 (5)	C13—O1—Sn1—C15	143.8 (3)
C20—C15—C16—C17	0.1 (5)	C13—O1—Sn1—O3	28.4 (5)
Sn1—C15—C16—C17	175.7 (3)	C13—O1—Sn1—N1	22.4 (3)
C15—C16—C17—C18	−0.8 (7)	C26—C21—Sn1—O1	173.8 (3)
C16—C17—C18—C19	0.3 (8)	C22—C21—Sn1—O1	−10.7 (3)
C17—C18—C19—C20	0.9 (8)	C26—C21—Sn1—C15	−87.7 (3)
C18—C19—C20—C15	−1.6 (7)	C22—C21—Sn1—C15	87.8 (3)
C16—C15—C20—C19	1.1 (6)	C26—C21—Sn1—O3	12.5 (3)
Sn1—C15—C20—C19	−174.7 (3)	C22—C21—Sn1—O3	−172.0 (3)
C26—C21—C22—C23	−0.2 (5)	C26—C21—Sn1—N1	87.6 (3)
Sn1—C21—C22—C23	−175.8 (3)	C22—C21—Sn1—N1	−96.9 (3)
C21—C22—C23—C24	−0.9 (6)	C20—C15—Sn1—O1	148.8 (3)
C22—C23—C24—C25	0.9 (7)	C16—C15—Sn1—O1	−26.8 (3)
C23—C24—C25—C26	0.1 (7)	C20—C15—Sn1—C21	48.6 (3)
C22—C21—C26—C25	1.3 (5)	C16—C15—Sn1—C21	−127.0 (3)
Sn1—C21—C26—C25	176.8 (3)	C20—C15—Sn1—O3	−52.3 (3)
C24—C25—C26—C21	−1.2 (6)	C16—C15—Sn1—O3	132.1 (3)
N6—C27—C28—C29	−0.7 (8)	C20—C15—Sn1—N1	−126.7 (3)
C27—C28—C29—C30	0.7 (7)	C16—C15—Sn1—N1	57.7 (3)
C28—C29—C30—C31	−0.5 (6)	C6—O3—Sn1—O1	−0.4 (4)
C28—C29—C30—C32	178.3 (4)	C6—O3—Sn1—C21	126.2 (2)
C29—C30—C31—N6	0.2 (7)	C6—O3—Sn1—C15	−115.5 (2)
C32—C30—C31—N6	−178.5 (4)	C6—O3—Sn1—N1	5.8 (2)
C31—C30—C32—O4	−3.3 (5)	C7—N1—Sn1—O1	−8.3 (3)
C29—C30—C32—O4	178.0 (3)	N2—N1—Sn1—O1	172.5 (2)
C31—C30—C32—N3	175.3 (4)	C7—N1—Sn1—C21	85.9 (3)
C29—C30—C32—N3	−3.5 (5)	N2—N1—Sn1—C21	−93.3 (3)
N4—C33—C34—C39	0.6 (6)	C7—N1—Sn1—C15	−99.0 (3)
N4—C33—C34—C35	178.7 (4)	N2—N1—Sn1—C15	81.8 (3)
C39—C34—C35—C36	−0.1 (6)	C7—N1—Sn1—O3	174.1 (3)
C33—C34—C35—C36	−178.3 (4)	N2—N1—Sn1—O3	−5.0 (2)
C34—C35—C36—C37	0.1 (6)	C39—O5—Sn2—C47	−103.9 (3)
C35—C36—C37—C38	−0.4 (6)	C39—O5—Sn2—C41	137.4 (3)
C36—C37—C38—O6	179.8 (3)	C39—O5—Sn2—O4	26.1 (5)
C36—C37—C38—C39	0.6 (6)	C39—O5—Sn2—N4	8.3 (3)
C35—C34—C39—O5	−179.7 (3)	C48—C47—Sn2—O5	−109.5 (3)
C33—C34—C39—O5	−1.6 (5)	C52—C47—Sn2—O5	70.5 (3)
C35—C34—C39—C38	0.3 (5)	C48—C47—Sn2—C41	−8.4 (4)
C33—C34—C39—C38	178.4 (3)	C52—C47—Sn2—C41	171.6 (3)
O6—C38—C39—O5	0.2 (4)	C48—C47—Sn2—O4	89.0 (3)
C37—C38—C39—O5	179.4 (3)	C52—C47—Sn2—O4	−91.0 (3)
O6—C38—C39—C34	−179.8 (3)	C48—C47—Sn2—N4	163.2 (3)
C37—C38—C39—C34	−0.6 (5)	C52—C47—Sn2—N4	−16.8 (3)
C46—C41—C42—C43	−0.5 (6)	C46—C41—Sn2—O5	−1.4 (3)
Sn2—C41—C42—C43	−178.4 (3)	C42—C41—Sn2—O5	176.4 (3)
C41—C42—C43—C44	−0.5 (7)	C46—C41—Sn2—C47	−105.4 (3)
C42—C43—C44—C45	0.3 (9)	C42—C41—Sn2—C47	72.4 (3)
C43—C44—C45—C46	0.7 (10)	C46—C41—Sn2—O4	155.9 (3)
C42—C41—C46—C45	1.5 (6)	C42—C41—Sn2—O4	−26.2 (3)
Sn2—C41—C46—C45	179.4 (4)	C46—C41—Sn2—N4	84.6 (3)
C44—C45—C46—C41	−1.7 (9)	C42—C41—Sn2—N4	−97.5 (3)
C52—C47—C48—C49	1.0 (7)	C32—O4—Sn2—O5	−21.6 (4)
Sn2—C47—C48—C49	−179.0 (4)	C32—O4—Sn2—C47	109.0 (3)
C47—C48—C49—C50	−2.0 (8)	C32—O4—Sn2—C41	−133.2 (2)
C48—C49—C50—C51	1.8 (9)	C32—O4—Sn2—N4	−3.2 (2)
C49—C50—C51—C52	−0.6 (8)	C33—N4—Sn2—O5	−8.6 (3)
C50—C51—C52—C47	−0.5 (8)	N3—N4—Sn2—O5	175.7 (3)
C48—C47—C52—C51	0.3 (7)	C33—N4—Sn2—C47	89.7 (3)
Sn2—C47—C52—C51	−179.7 (3)	N3—N4—Sn2—C47	−86.1 (3)
C8—C7—N1—N2	177.0 (3)	C33—N4—Sn2—C41	−100.1 (3)
C8—C7—N1—Sn1	−2.1 (5)	N3—N4—Sn2—C41	84.2 (3)
O3—C6—N2—N1	2.2 (5)	C33—N4—Sn2—O4	179.0 (3)
C4—C6—N2—N1	−177.4 (3)	N3—N4—Sn2—O4	3.2 (2)
